# Engineered Protein Copolymers for Heparin Neutralization and Detection

**DOI:** 10.1021/acs.biomac.2c01464

**Published:** 2023-01-04

**Authors:** Qing Liu, Ahmed Shaukat, Zhuojun Meng, Sami Nummelin, Tekla Tammelin, Eero Kontturi, Renko de Vries, Mauri A. Kostiainen

**Affiliations:** † Biohybrid Materials, Department of Bioproducts and Biosystems, Aalto University, Aalto00076, Finland; ‡ Wenzhou Institute, University of Chinese Academy of Sciences (WIUCAS), Wenzhou325001, China; § Materials Chemistry of Cellulose, Department of Bioproducts and Biosystems, Aalto University, Aalto00076, Finland; ∥ VTT Technical Research Centre of Finland Ltd, VTT, P.O. Box 1000, EspooFI-02044, Finland; ⊥ Physical Chemistry and Soft Matter, Wageningen University and Research Centre, Wageningen6708 WE, The Netherlands

## Abstract

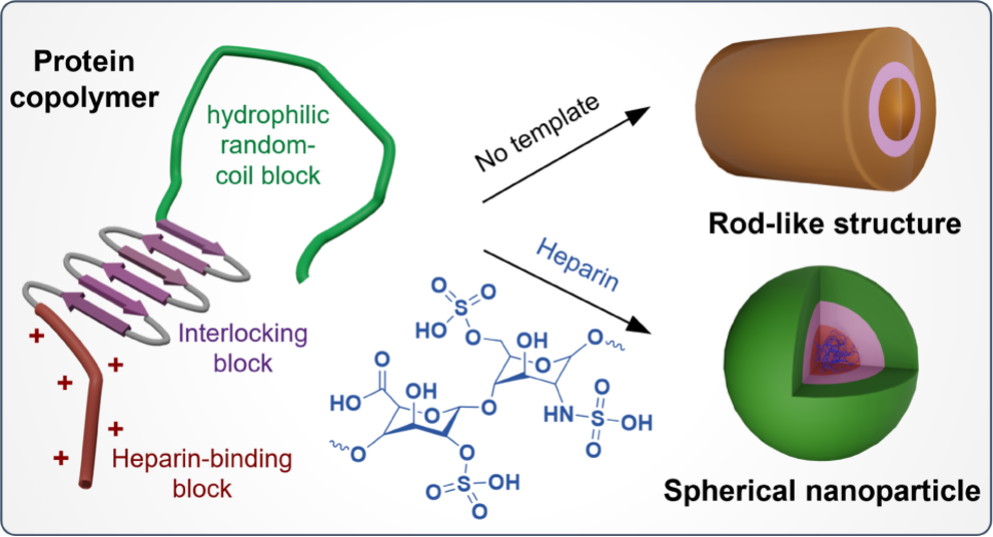

Heparin is a widely applied anticoagulant agent. However, in clinical practice, it is of vital importance to reverse its anticoagulant effect to restore the blood-clotting cascade and circumvent side effects. Inspired by protein cages that can encapsulate and protect their cargo from surroundings, we utilize three designed protein copolymers to sequester heparin into inert nanoparticles. In our design, a silk-like sequence provides cooperativity between proteins, generating a multivalency effect that enhances the heparin-binding ability. Protein copolymers complex heparin into well-defined nanoparticles with diameters below 200 nm. We also develop a competitive fluorescent switch-on assay for heparin detection, with a detection limit of 0.01 IU mL^–1^ in plasma that is significantly below the therapeutic range (0.2–8 IU mL^–1^). Moreover, moderate cytocompatibility is demonstrated by *in vitro* cell studies. Therefore, such engineered protein copolymers present a promising alternative for neutralizing and sensing heparin, but further optimization is required for *in vivo* applications.

## Introduction

Heparin is one of the most important naturally derived anticoagulant agents. Its medicinal role originates from the ability to bind coagulant factors and inhibit the blood-clotting cascade.^1−3^ However, severe side effects can be triggered by the overdosage of heparin, including thrombocytopenia, hemorrhage, and osteoporosis.^4,5^ Therefore, it is essential to monitor and neutralize excess heparin. To date, anti-Xa assay and activated partial thromboplastin time assay are the most prevalent techniques in monitoring heparin concentration. However, improvements are still needed, including detection efficiency and consistency.^6^ Välimäki et al. designed a dye displacement assay for the quantification of heparin by measuring the absorption change of the dye.^7^ Though heparin detection in 10% plasma was demonstrated, the sensitivity was beyond satisfactory. Chen et al. synthesized a tetraphenylethylene-coupled metallacycle that was applied to detect heparin via aggregation-induced emission.^8^ However, the biocompatibility of the sensor needed further demonstration, and the laborious preparation might restrict it from widespread applications.

As for heparin-reversal agents, protamine sulfate (PS) is the only antidote licensed for heparin over the last 80 years.^9,10^ Though its efficiency has been proven, serious adverse effects, such as systemic hypotension and bronchospasm, caused by PS and PS–heparin complex have risen major concerns.^10,11^ Therefore, continuous efforts are being dedicated to the discovery of novel alternatives with improved efficiency and reduced adverse effects. The proposed alternatives include, but are not limited to, small molecules,^12,13^ (bio)polymers,^14−17^ and self-assembled systems.^18−21^ Though their performance in neutralizing heparin has been demonstrated, there are also selected limitations. For instance, the heparin-neutralizing ability of small molecules can be significantly compromised in biological media,^22^ which can, however, be circumvented by using higher-molecular-weight (bio)polymers. However, their synthesis is not as straightforward and polymers with high dispersity can result in batch-to-batch performance variations.^14^ In the case of self-assembled systems, the *in vivo* fate of the building blocks still requires clarification.^21^ Therefore, a safe and efficient heparin-neutralizing agent is still highly demanded. A particularly intriguing category is the sophisticated candidates that can compact and sequester heparin into small and biologically inert particles.^16,23^

In nature, precise compaction is a fundamental process in all life forms. For example, the genetic materials are condensed by histone proteins into chromosomes in most eukaryotic organisms.^24−27^ In viruses, the genome is sequestered and protected by protein capsids, a process that is highly controlled by specific interactions.^28,29^ Subsequent research revealed that the genetic materials are not crucial for the capsid formation, and noncognate polyelectrolytes can be loaded during the capsid formation or by diffusion through the pores on selected capsids.^30−32^ Such a delicate mechanism has stimulated researchers to discover promising heparin antidote that can neutralize heparin via a similar inclusion–sequestration process in an attempt to minimize potential side effects.^14,20,21^ Recombinant proteins can serve as a strong candidate to fulfill this task. Compared to their polymeric counterparts, recombinant proteins are more biocompatible and can be produced on large scale yet with arbitrary structural design and precise size control.^33^ Hernandez-Garcia et al. constructed a series of artificial protein copolymers (PCSs) and realized precise encapsulation of diverse DNA structures.^34,35^ Particularly, they found that the introduction of a silk-like sequence could trigger a progressive binding to linear DNA templates and eventually lead to the formation of rod-shaped virus-like particles, a process that highly mimicked the self-assembly kinetics of tobacco mosaic virus.^36,37^

In this work, we produced three PCSs and evaluated their performance in neutralizing and sensing heparin. We demonstrated the importance of the silk-like sequence in enhancing the heparin-neutralization ability. PCSs complexed heparin into nanoparticles with a narrow size distribution, which was in contrast to the PS–heparin aggregates. We also developed a competitive fluorescence switch-on assay for heparin detection, and an ultralow limit of detection (0.01 IU mL^–1^) in plasma was achieved, which is significantly below the used therapeutic dosages (0.2–8 IU mL^–1^).^38^ Therefore, these PCSs are anticipated to make a promising neutralizing and detecting agent for heparin.

## Experimental Section

### Materials

All chemicals were purchased from commercial suppliers and used without further purification. Heparin, methylene blue, citric acid, Dulbecco’s modified Eagle’s medium (DMEM), fetal bovine serum (FBS), penicillin, 3-(4,5-dimethylthiazol-2-yl)-2,5-diphenyltetrazolium bromide (MTT), and blood plasma were ordered from Sigma-Aldrich. Two-stage heparin assay kit, Biophen Anti-Xa (221005), was ordered from HYPHEN BioMed. Red blood cells (RBCs) were ordered from Cambridge Bioscience Ltd. Fluorescein (6-FAM) and black hole quencher (BHQ1)-modified 30 nucleotide (nt) DNA (sequence: 5′-FAM-TTT TTT TTT TTT TTT TTT TTT TTT TTT TTT-BHQ-3′) was ordered from Integrated DNA Technology.

### Protein Copolymer Preparation and Characterization

Three protein copolymers (PCS0, PCS4, and PCS10) were prepared and characterized according to the reported procedures.^36^ Successful production was verified by sodium dodecyl sulfate–polyacrylamide gel electrophoresis (SDS-PAGE) and matrix-assisted laser desorption ionization time-of-flight mass spectrometry (MALDI-TOF MS, Figure S1).

### AFM Imaging

For atomic force microscopy (AFM) imaging, a 5 μL droplet of PCS10 or protein copolymer–heparin complexes in PB or PBS was deposited on a freshly cleaved mica substrate. Heparin in both media was 0.1 mg mL^–1^. Protein copolymers were used to fully neutralize heparin (phosphate buffer (PB)/PCS0 = 4 mg mL^–1^, PCS4 = 5 mg mL^–1^, PCS10 = 7 mg mL^–1^, and PS = 0.2 mg mL^–1^; phosphate buffered saline (PBS)/PCS0 = PCS4 = PCS10 = 10 mg mL^–1^ and PS = 0.3 mg mL^–1^). PCS10 with the same concentration in the absence of heparin was also imaged accordingly. The sample was incubated on mica for 5–15 min, subsequently dipped in HPLC-grade water, and dried in a stream of ultrapure air. AFM imaging was performed in the air using Agilent 5100 AFM in intermittent contact mode and HQ:NSC18/Al BS cantilevers from MikroMasch.

### Cryo-TEM

The cryogenic transmission electron microscopy (cryo-TEM) images were collected using a JEM 3200FSC field emission microscope (JEOL) operated at 300 kV in bright-field mode with an Omega-type zero-loss energy filter. The images were acquired with Gatan Digital Micrograph software, while the specimen temperature was maintained at −187 °C. The cryo-TEM samples were prepared by placing 3 μL aqueous dispersion of PCS10 on a 200 mesh Lacey carbon film on Copper TEM Grids (Agar Scientific) and plunge-frozen into liquid ethane using a Leica grid plunger with 3 s blotting time under 100% humidity. The grids with vitrified sample solution were maintained at liquid nitrogen temperature and then cryo-transferred to the microscope. The TEM grids were plasma cleaned before use (NanoClean 1070, Fischione Instruments).

### Anti-Xa Assay

Heparin neutralization with compounds was evaluated using a commercial two-stage kit, Biophen Anti-Xa (221005). Protein copolymers of different concentrations were first lyophilized and redissolved in plasma. Dissolved compounds were then added to the heparin solution in 150 mM NaCl (0.1 mg mL^–1^), giving a final heparin concentration of 0.045 IU mL^–1^ (12.5 nM) and protein copolymer/heparin mass ratio from 0 to 150 for all protein copolymers. Kit reagents were utilized according to the manufacturer’s instructions. To run the calorimetric assay, 40 μL of the protein copolymer–heparin solution was added to a 96-well microplate followed by the addition of 40 μL of antithrombin and incubation for 2 min. Then, 40 μL of factor Xa was added and incubated for another 2 min. Afterward, 40 μL of the factor-Xa-specific chromogenic substrate was added to the solution and left to react for 2 min. Finally, the reaction was quenched by introducing 80 μL of 2% citric acid. The absorbance at 405 nm was recorded immediately using a BioTek Cytation three-microplate reader. The anticoagulant activity is inversely proportional to the measured absorption intensity, and the percentage of neutralization was determined using a calibration curve constructed according to the manufacturer’s instructions (Figure S4). Measurements were performed using triplicate samples.

### DLS Measurement

The dynamic light scattering (DLS) measurements were carried out with a Zetasizer Nano ZS device (Malvern Instruments) with a 4 mW He–Ne ion laser at the wavelength of 633 nm and an avalanche photodiode detector at an angle of 173°. Zetasizer software (Malvern Instruments) was used to attain the data. Cumulant analysis was used to obtain the intensity mean value of the complex size, that is, the hydrodynamic diameter. Experiments were carried out at 25 °C. Heparin solutions were prepared by diluting 10 mg mL^–1^ heparin stock solution into 0.01 mg mL^–1^ in 0.3 mL of the buffer. The heparin solutions were titrated with 2 μL of sample solutions (different protein copolymer concentrations) resulting in a total sample volume of 20 μL. Measurements were carried out in PB or PBS. After every addition, the samples were allowed to equilibrate for 1 min. Each titration series was carried out three times, and all titration points were measured three times.

### QCM-D

Interaction between surface-immobilized heparin and PCS or PS was investigated by using gold-coated sensors and a quartz crystal microbalance with dissipation monitoring (QCM-D) unit (E4 instrument, Q-Sense AB, Sweden). The sensors were first cleaned with UV/ozone treatment for 15 min, followed by immersion in a 0.1 wt % polyethyleneimine (PEI) for 30 min to absorb a PEI layer. Afterward, the PEI-coated sensors were thoroughly rinsed with Milli-Q water and dried with nitrogen gas. Heparin coverage was performed *in situ* to establish irreversible binding and full surface coverage before binder solution injection. After reaching a stable baseline with heparin solution, a buffer solution was applied to rinse and remove loosely bound molecules. Finally, the binder solutions were applied, and the shifts in dissipation and frequency were monitored. All binders were dissolved in PB or PBS to yield a 0.1 mg mL^–1^ concentration. All solutions were filtered by using 0.45 μm filters before tests. Experiments were performed at a constant flow rate of 20 μL min^–1^, and the temperature was maintained at 23 °C.

### Switch-On Heparin Detection

The quenching effect of protein copolymers on FAM- and BHQ-modified DNA (30 nt) was evaluated by titrating 0.1 μM DNA with concentrated protein copolymers. The fluorescence intensity plateau was reached at 15, 30, and 60 μg mL^–1^ for PCS0, PCS4, and PCS10, respectively, in PB, while those in PBS were 500, 400, and 270 μg mL^–1^, respectively. The concentrations were then applied to fully quench DNA (0.1 μM) for the subsequent heparin titration in corresponding buffers. The fluorescence intensity plateau was reached at 700 μg mL^–1^ for PCS10 in plasma, and the concentration was then applied to fully quench DNA for the subsequent heparin titration. The plasma volume (70%) was kept constant in all the samples.

### Hemolysis Assay

The detailed procedure for the hemolysis assay has been previously reported.^39^ Generally, RBCs were purchased from Cambridge Bioscience Ltd. (U.K.) and stored at 4 °C. Before samples were added, 1 mL of blood was centrifuged at 500×*g* for 5 min and the plasma was removed gently. The remaining RBCs were washed with 1× PBS three times and redispersed to the initial volume in 1× PBS. The cells were diluted 50× and split into 96-well culture plates (190 μL/well). The concentrated protein copolymer (10 μL) or protein copolymer–heparin solutions in 1× PBS were added to each well, resulting in the desired final protein copolymer concentrations (100–500 μg mL^–1^). 10 μL of 20% Triton X-100 in 1× PBS and 10 μL of 1× PBS were added as positive and negative controls, respectively. After incubation at 37 °C for 1 h, the plates were centrifuged for 5 min at 500×*g* to pellet intact erythrocytes, and 100 μL of the supernatant from each well was delicately transferred into a clear 96-well plate. The resulting hemoglobin in the supernatant was measured at 540 nm with a microplate reader (Cytation 3, BioTek). The percentage of hemolysis was calculated as follows
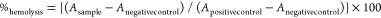


The measurements were performed using triplicate samples.

### Cell Culture and MTT Dye Assay

Human dermal fibroblasts (HDF) and HepG2 cells were purchased from Fisher Scientific and used to evaluate the cytocompatibility of binders. The cells were then expanded in DMEM substituted with 10% FBS, 100 U mL^–1^ penicillin, and 100 μg mL^–1^ streptomycin. The cells were kept in humidified conditions with 5% CO_2_ at 37 °C. Once 90% confluency was reached, the cells were split using 0.25% ethylenediaminetetraacetic acid–trypsin. Cell passages between 3 and 5 were used for the cell culture studies. Before the MTT assay, cells were split into 96-well culture plates (approximately 10,000 cells/well) and incubated for 24 h. After the incubation, the culture media were replaced with 100 μL of protein copolymer solutions (0.1–100 μg mL^–1^) in DMEM supplemented with 10% FBS, 100 U mL^–1^ penicillin, and 100 μg mL^–1^ streptomycin. The cells were then kept in humidified conditions with 5% CO_2_ at 37 °C for 14 or 24 h. After that, the sample solutions in each well were replaced with 100 μL of complete media and 10 μL of MTT solution (5 mg mL^–1^ in PBS). After 4 h of incubation at 37 °C with 5% CO_2_, the MTT solution was replaced with 100 μL DMSO in each well to dissolve formazan crystals before reading. The absorbance was measured with a microplate reader (Cytation 3, BioTek) at the wavelength of 570 nm. Measurements were carried out using triplicate samples.

### Statistical Analysis

All data are shown as mean ± standard deviation. Statistical values are indicated in figures according to: * indicates *p* < 0.05 and ** indicates *p* < 0.01.

## Results and Discussion

**Figure 1:**
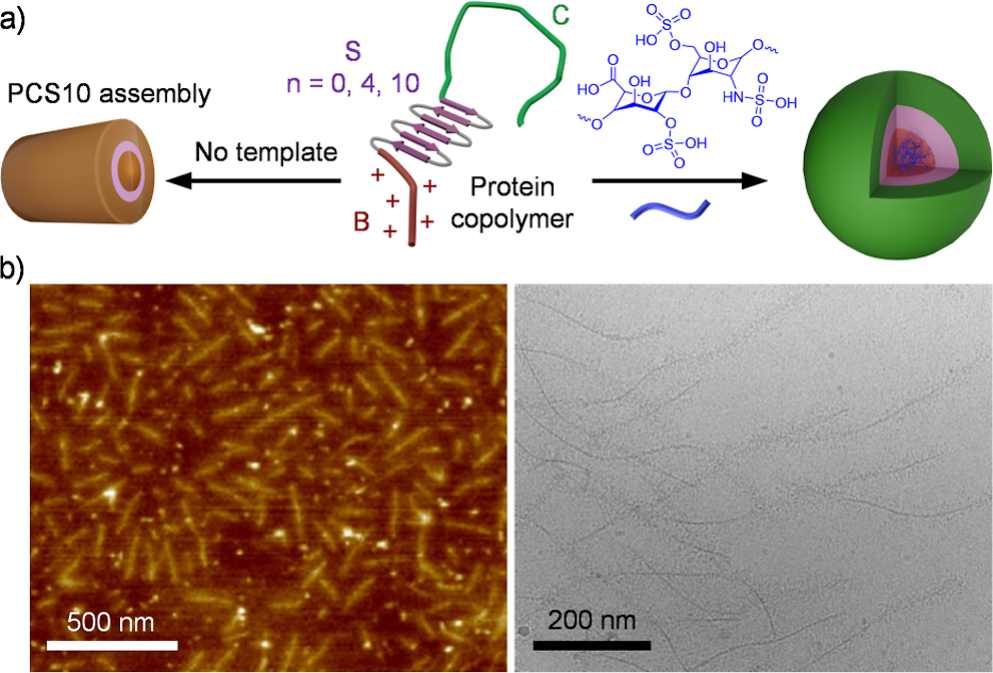
Structural presentation of PCSs. (a) Schematic illustration of the elementary blocks of PCSs and the assemblies with or without heparin. In the single PCS model, B represents the N-terminal dodecalysine (K_12_) heparin-binding segment (red), C represents a C-terminal hydrophilic random-coil block that contains 407 amino acids (green), and S represents a silk-like sequence with varying repeating units [pink, S: (GAGAGAGQ)_*n*_, *n* = 0, 4, or 10]. (b) AFM (left) and cryo-TEM (right) images of PCS10.

Three PCSs were prepared, and they contained two elementary blocks: a cationic dodecalysine-binding block (B: K_12_) and a hydrophilic random-coil block (C: ∼400 amino acids) (Figure 1a and Table S1). The oligolysine block was reported to effectively bind DNA substrates via nonspecific electrostatic interactions while the random-coil block was designed to be inert to the environment and to prevent aggregation.^36^ A series of precisely tuned silk-like sequence (S: (GAGAGAGQ)_*n*_, *n* = 0, 4, or 10, Figure 1a) was inserted between the binding block and the isolating block separately, and the three PCSs are denoted as PCS0, PCS4, and PCS10 hereafter. The silk-like sequence could fold and stack into filamentous structures, which was anticipated to offer PCSs with controlled intermolecular interaction and consequently enhanced heparin-binding ability induced by the multivalency effect.^36,40^

The successful production and purity were verified by SDS-PAGE and MALDI-TOF MS (Figure S1). As expected, plain PCS10 self-assembled into rod-like particles with a diameter of ∼20 nm and lengths of hundreds of nanometers, a structure that was mediated by the cooperative interaction between the silk-like sequence (Figure 1b).^36^

Before we proceeded to evaluate the *in vitro* performance of PCSs in application-relevant media (150 mM NaCl), the heparin-reversal ability in the absence of salt was measured. Initial tests were carried out in a phosphate buffer (PB: 10 mM Na_2_HPO_4_–NaH_2_PO_4_, pH = 7.4) by methylene blue (MB) displacement assay, and the heparin-neutralization performance was plotted as a function of the molar ratio of binder to heparin (*n*_B_/*n*_H_).^41^ As can be seen from Figure S2, heparin was gradually bound with increasing binder concentrations until a saturation point was reached, indicating that all heparin could be neutralized by binders in the absence of salt. The amount of PCSs that was required to reach the binding plateau increased with the repeating units of the silk-like sequence (molar ratio at saturation point: PCS0 and PCS4: ∼19; and PCS10: ∼28). In order to assess the heparin-binding efficiency, we calculated the effective concentration (EC_50_) that was required to achieve 50% heparin neutralization and the corresponding charge ratio (ξ) of all binders (Table 1).^18^ PCS0 had an EC_50_ value of 100 ± 3 nM and a ξ value of 0.36 ± 0.01, which were lower than those of PCS4 (153 ± 14 nM and 0.56 ± 0.05) and PCS10 (241 ± 8 nM and 0.87 ± 0.03). We attribute the excellent binding efficiency (ξ below 1 for all binders) to the polyelectrolyte nature, which generated an intramolecular multivalency effect that enhanced the binding efficiency.^40^ On the other hand, the intermolecular interaction induced by the silk-like sequence hindered the mobility of the oligolysine segment, which compromised the binding efficiency of PCS4 and PCS10.

**Table 1:** Derived Data from MB Binding Assay and Anti-Xa Assay

		MB Displacement Assay (PB)^a^		Anti-Xa Assay (PBS)^b^	
Binder	Nominal charge	EC_50_ (nM)^c^	ξ^d^	EC_50_ (nM)^c^	ξ^d^
PS	+24^e^	47 ± 2	0.51 ± 0.02	5 ± 1	0.46 ± 0.02
PCS0	+12	100 ± 3	0.36 ± 0.01	>437^f^	>7^f^
PCS4		153 ± 14	0.56 ± 0.05	29 ± 1	0.93 ± 0.03
PCS10		241 ± 8	0.87 ± 0.03	26 ± 1	0.83 ± 0.01

a MB displacement assay condition (heparin: 1 μg mL^–1^).

b Anti-Xa assay condition (heparin: 0.045 IU mL^–1^).

c EC_50_ represents the required binder concentration for 50% heparin neutralization.

d ξ indicates the charge ratio between binder and heparin at the corresponding 50% heparin neutralization.

e The assumed positive charges of PS.^18^

f The maximum amount used in the assay.

The successful heparin-binding performance was confirmed by the changes in hydrodynamic diameters acquired from DLS measurements (Figure S3a,b). The light-scattering intensity (derived count rates) was also obtained as an indicator for heparin binding and complex formation (Figure S3c).^14,15^ All PCSs formed nanoscale complexes with heparin, and the sizes were uniform and below 150 nm (Figure S3a). AFM images also revealed the well-dispersed PCS–heparin complexes (Figure S3d), which were distinct from the fibrous structures formed by heparin and protein copolymers containing heparin-binding KRSR domains.^42^ On the contrary, complexing PS with heparin yielded a typical coacervate structure, as evidenced by the AFM image and the growing particle size (Figure S3a), as well as the emergence of a peak in the derived count rate (Figure S3c) from the DLS measurement.^43^

**Figure 2:**
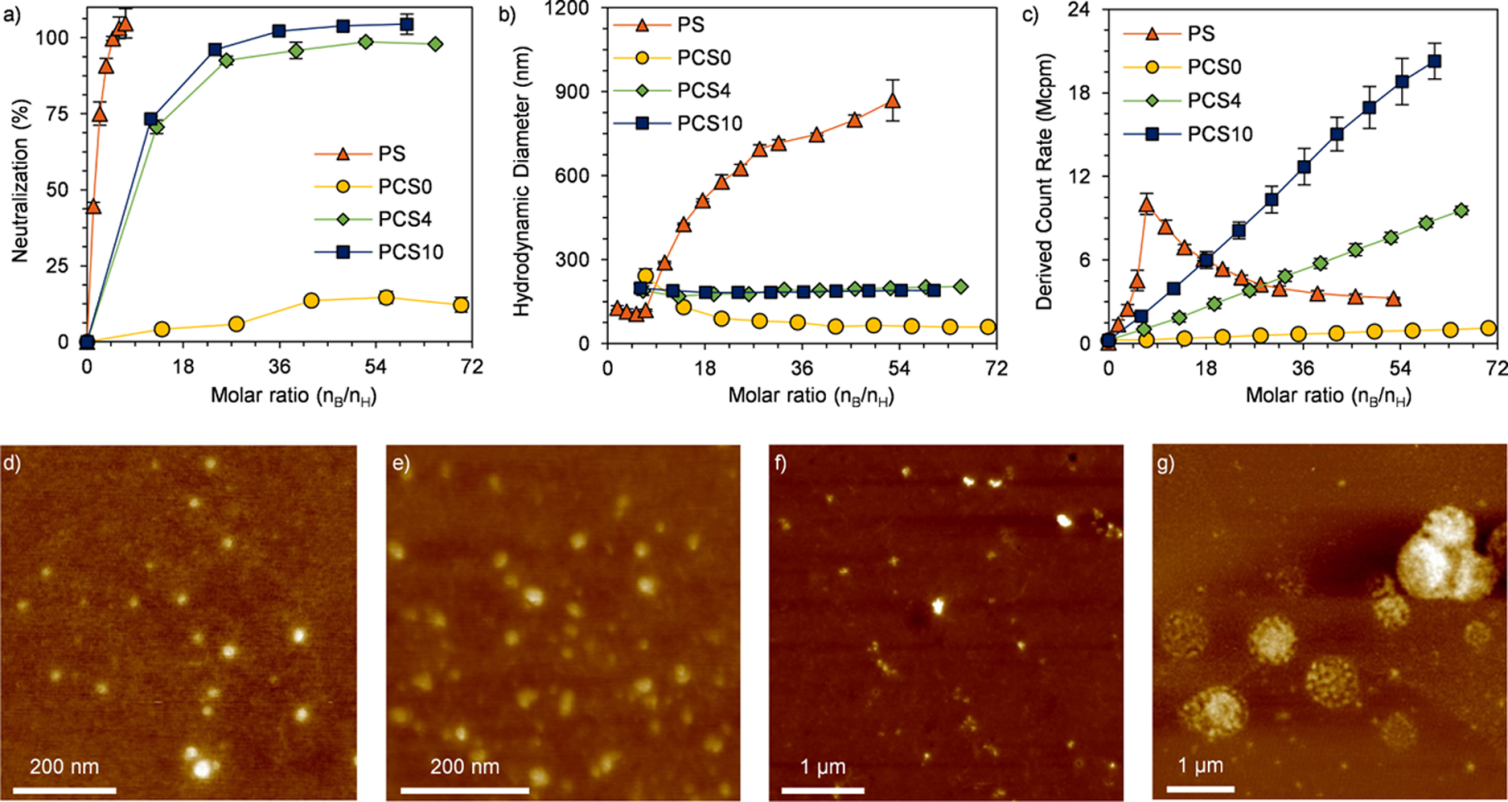
Evaluation of the heparin neutralization ability of binders in PBS. (a) Heparin neutralization performance measured with the anti-Xa assay. Heparin concentration: 0.045 IU mL^–1^; (b,c) hydrodynamic diameter (b) and derived count rate (c) obtained from DLS measurements in PBS. Heparin concentration: 0.01 mg mL^–1^. Data are presented with means ± standard error of the mean (S.E.M.) (*n* = 3); (d–g) AFM images of heparin complexed with PCS0 (d), PCS4 (e), PCS10 (f), and PS (g) in PBS.

Successful complexation between heparin and binders under application-relevant conditions (PBS: PB, 150 mM NaCl) was verified by a chromogenic anti-Xa assay and DLS measurements (Figure 2a–c and S3e). From the anti-Xa assay result, we could observe that the interaction between PCS0 and heparin was significantly screened by salt, and less than 15% heparin was neutralized (Figure 2a). On the other hand, complete neutralization was reached with PCS4 and PCS10. For PCS4, the molar ratio at the saturation point increased to ∼40 compared to ∼19 derived from MB displacement assay, and that of PCS10 remained almost unchanged (∼30). From the derived data summarized in Table 1, we found that the charge efficiency (ξ) of PCS4 was elevated by the electrolyte, implying the decreased efficiency in neutralizing heparin. On the other hand, the ξ values of PCS10 and PS were slightly lower than those derived from MB displacement assay (from 0.87 ± 0.03 to 0.83 ± 0.01 for PCS10 and from 0.51 ± 0.02 to 0.46 ± 0.02 for PS), a phenomenon that was observed before.^18^ We attributed the behavior of PCS10 to the multivalency effect induced by the long silk-like interlocking segment and the increased mobility of the oligolysine segment caused by the screening effect of NaCl.^40^

DLS measurement results (Figures 2b,c and S3e) showed that all the binders exhibited the same behavior as those in PB: while complexes below 200 nm were obtained by PCSs and heparin in the whole molar ratio, PS complexed with heparin into micron-sized structures and the size increased with PS concentration. The morphologies of all binder–heparin complexes were visualized under AFM (Figure 2d–g). All binder–heparin complexes adopted similar morphologies as those in PB. While the PCS–heparin complex maintained a compact structure in PBS, the PS–heparin complex was significantly influenced by electrolytes, as revealed by the loose configuration of the complex. The large and irregular PS–heparin coacervates have been proposed to be connected to the toxicity of PS.^11,44^ PCS–heparin nanoparticles, on the other hand, are more distinguished in size and surface properties, which is promising in mitigating systematic toxicity.^45^

**Figure 3:**
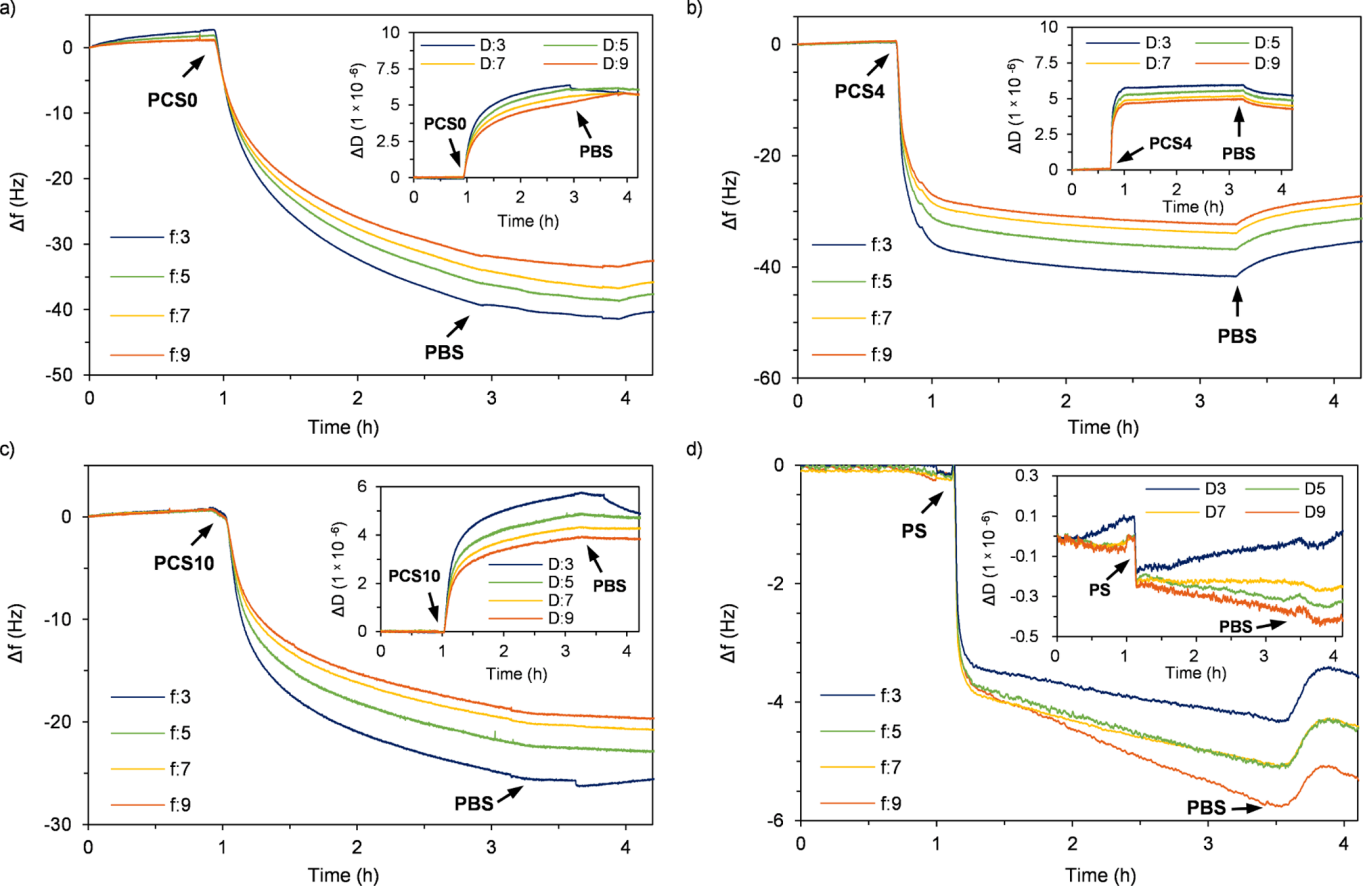
Frequency shifts (Δ*f*) and energy dissipation (Δ*D*, insets) graphs from QCM-D measurements on the evaluation of PCS0 (a), PCS4 (b), PCS10 (c), and PS (d) capturing heparin in PBS.

We also employed QCM-D to verify the heparin-binding ability under a constant flow. A gold sensor was first absorbed with PEI, and a second heparin layer was deposited *in situ* on the PEI layer. Subsequently, PCS or PS solutions were purged through the functionalized sensor, and the binding performance was evaluated by measuring changes in resonance frequency shift (Δ*f*) and energy dissipation (Δ*D*) as a function of time. As can be seen in Figures S3 and 3, all samples exhibited dramatic changes in Δ*f* when the sample solution was pumped in, proving the effective heparin-capturing ability under constant flows. The downward trajectory was less steep after the initial drop, which indicated that the heparin on the sensor was gradually saturated by binders. When the solution was switched to buffer again, a slight increase in frequencies was observed for all binders, implying that a small portion of the binders was washed away by the buffer.^41^ A compromised binding between heparin and PCS0 was also observed, as evidenced by a less sharp frequency drop in PBS compared to that in PB (Figures 3a and S5a). No obvious change was observed with PCS4 and PCS10, demonstrating the excellent heparin-capture ability even under physiologically relevant conditions. Additionally, positive changes in Δ*D* were observed with all PCSs (inserts in Figures 3 and S5), which confirmed the successful binding. The clear increase in energy dissipation indicated rheological changes toward viscoelastic behavior of the surface caused by the bound proteins.^14,41^ In the case of PS, no obvious change in energy dissipation revealed that the polyelectrolyte complexation had no obvious impact on the surface rigidity i.e., the surface displayed elastic behavior.^41^

**Figure 4:**
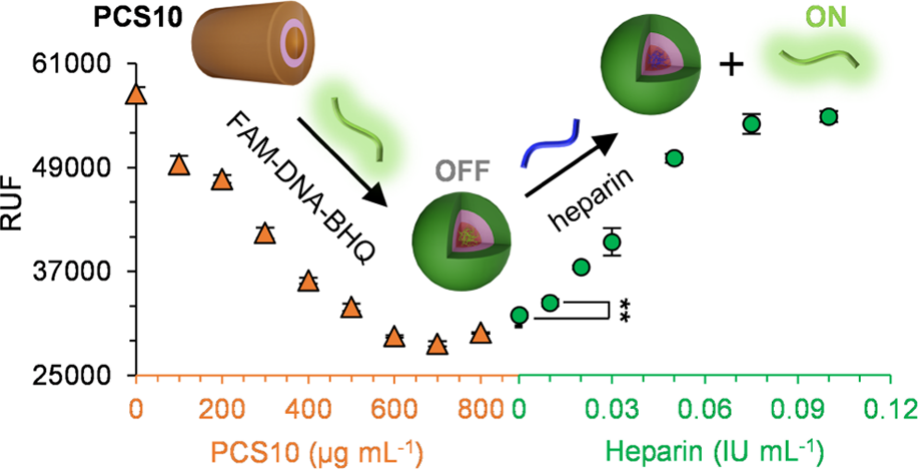
Fluorescent switch-on detection of heparin using PCS10 in plasma. The inset shows the schematic steps of the process. Orange triangles indicate the titration of modified DNA (0.1 μM) with PCS10. Green circles indicate the fluorescence recovery upon the addition of heparin to the PCS10–DNA complex. Data are presented with means ± standard error of the mean (S.E.M.) (*n* = 3). **p < 0.01.

Fluorescent probes are attractive in detecting heparin due to their sensitivity and low cost.^1^ Of particular interest is the fluorescence switch-on probes owing to their inertia to false targets.^46^ With the help of a chemically modified DNA (30-nt-long DNA-bearing 6-FAM and BHQ1 at the 5′ and 3′ ends, respectively), we designed a sensitive assay for the detection of heparin. As illustrated in the schematic in Figure 4, the fluorescent DNA chain was first complexed with PCS, leading to a reduced FAM–BHQ distance and a subsequent quenching in fluorescence intensity. When heparin was added, owing to its high charge density, DNA in the PCS–DNA complexes was displaced by heparin, yielding free DNA and consequently, a regain in fluorescence intensity. As shown in Figures S6–S8, PCSs significantly quenched the fluorescence of DNA with or without 150 mM NaCl, and a quenching plateau was reached. The binding between PCSs and DNAs was hindered by NaCl, and more proteins were required to fully complex DNA in PBS to reach a quenching plateau. When heparin was added to the PCS–DNA solution, the fluorescence intensity was gradually recovered and linearly correlated with heparin concentration until the original fluorescence intensity was restored. It was found that the detection limit was below the lowest therapeutic level (0.2 IU mL^–1^) for PCS4 and PCS10 under both conditions.^4^ More importantly, we evaluated the heparin detection performance in plasma for PCS10, and a detection limit of 0.01 IU mL^–1^ was realized (Figure 4), making PCS10 a promising heparin probe for future heparin-sensing applications.

**Figure 5:**
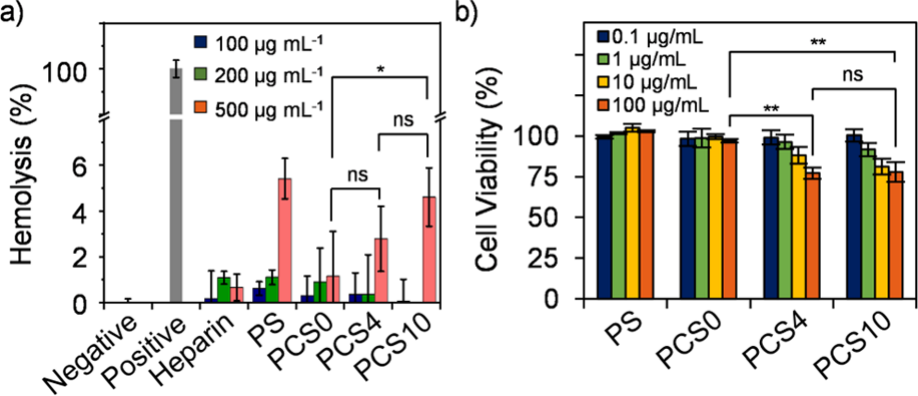
Evaluation of the cytocompatibility of heparin binders. (a) Hemolytic activity of binders on RBCs. Solutions of 1× PBS and 1% Triton X-100 were used as the negative and positive control, respectively; (b) effect of heparin binders on the HDF cell viability evaluated with MTT assay for 14 h. Data are presented with means ± standard error of the mean (S.E.M.) (*n* = 3). *p < 0.05, **p < 0.01.

A hemolysis assay and an MTT dye assay were applied to evaluate the cytocompatibility of PCSs. PS was also tested for comparison. As can be seen in Figure 5a, increased hemolytic activity was induced with an increasing concentration of binders, and about 5% of RBCs were hemolyzed when the concentration reached 500 μg mL^–1^, indicating cytocompatibility with RBCs. We noticed that increasing the silk-like sequence slightly increased the hemolytic effect on RBCs. MTT assay also indicated that increasing the silk-like sequence imposed an influence on the viability of dermal fibroblasts (HDF) cells and liver cancer cells, HepG2 (Figures 5b and S9).^15^ No obvious change in HDF cell viability was observed with PS or PCS0 up to 100 μg mL^–1^ after 24 h incubation. On the other hand, ∼80% of HDF cells remained alive with PCS4 and PCS10 up to 100 μg mL^–1^ in 14 h and the value dropped to ∼70% after 24 h incubation (Figure S9a). The measurement with HepG2 showed the same trend, and the cell viability was decreased to ∼60% for PCS4 and ∼50% for PCS10 in 14 h. The toxicity could be attributed to the silk-like segment or the increased positive charge density induced by the multivalency effect.

## Conclusions

Herein, inspired by virus capsid proteins, we designed and prepared three artificial PCSs for efficient heparin neutralization and detection. The as-prepared PCSs were engineered with a heparin-binding block, an interlocking block with varying repeating units, and an isolating block. Their efficiency in encapsulating heparin into well-defined complexes was demonstrated. In particular, we found that cooperative interprotein binding could be achieved by carefully tuning the size of the interlocking block. The cooperative binding was demonstrated to produce a multivalency effect, which played a key role in stabilizing the PCS–heparin complexes under physiologically relevant conditions. A competitive fluorescence switch-on assay was developed with the help of chemically modified DNA, and an ultralow heparin-probing limit (0.01 IU mL^–1^) that is well below the therapeutic dosage (0.2–8 IU mL^–1^) was achieved in plasma. Moreover, moderate cytocompatibility was demonstrated. Nevertheless, optimization over the binding efficiency and cytocompatibility is still needed in future work, which is feasible via the recombinant approach. For example, the binding block with an increased length can be engineered, which is anticipated to enhance the binding ability, as well as the cytocompatibility due to the reduced amount of binder that is required. Therefore, combined with other merits (structural precision, large-scale production, etc.), such recombinant proteins can serve as promising candidates in reversing and sensing heparin.

## Supporting Information Available

Procedures for SDS-PAGE, MALDI-TOF MS, and MB displacement assay; full amino acid sequences of the three PCSs; SDS-PAGE image and MALDI-TOF MS for PCSs; MB displacement assay results; DLS measurement results and AFM images in PB; a calibration curve for anti-Xa assay; QCM-D measurement results in PB; fluorescence switch-on detection results in PB and PBS for all PCSs; and MTT assay results of the incubation with HDF for 24 h and HepG2 for 14 h (PDF)
