# Supplementary material for: Engineered Protein Copolymers for Heparin Neutralization and Detection
Source: Biomacromolecules. 2023 Jan 4;24(2):1014–21. doi: 10.1021/acs.biomac.2c01464 (PMC9930113; doi:10.1021/acs.biomac.2c01464)
Supplement: Supplementary file 1 — bm2c01464_si_001.pdf [file bm2c01464_si_001.pdf]

Supporting Information

Engineered Protein Copolymers for Heparin Neutralization and Detection

*Qing Liu, Ahmed Shaukat, Zhuojun Meng, Sami Nummelin, Tekla Tammelin, Eero Kontturi, Renko de Vries, Mauri A. Kostiainen**

Qing Liu, Ahmed Shaukat, Sami Nummelin, Mauri A. Kostiainen

Biohybrid Materials, Department of Bioproducts and Biosystems, Aalto University, 00076 Aalto, Finland.

Qing Liu, Zhuojun Meng

Wenzhou Institute, University of Chinese Academy of Sciences (WIUCAS), Wenzhou 325001, China.

Zhuojun Meng, Eero Kontturi

Materials Chemistry of Cellulose, Department of Bioproducts and Biosystems, Aalto University, 00076 Aalto, Finland.

Tekla Tammelin

VTT Technical Research Centre of Finland Ltd, VTT, PO Box 1000, FI-02044 Espoo, Finland.

Renko de Vries

Physical Chemistry and Soft Matter, Wageningen University and Research Centre, Wageningen, The Netherlands.

Characterization

Sodium dodecyl sulfate-polyacrylamide gel electrophoresis (SDS-PAGE). SDS-PAGE was carried out using the NuPAGE Novex system (Invitrogen, Carlsbad, CA) with 10% Bis-Tris gels, MES SDS as running buffer, and SeeBlue Plus2 prestained molecular mass markers. 10 μL of protein solutions (0.1 mg mL^{-1}) were run. Gels were stained with Coomassie SimplyBlue SafeStain (Invitrogen).

Matrix-assisted laser desorption ionization time-of-flight mass spectrometry (MALDI-TOF MS). MALDI-TOF MS was carried out in an Ultraflex mass spectrometer (Bruker, Billerica, MA). Protein samples were prepared by the dried droplet method. The matrix was made up of 5 mg mL^{-1} 2.5-dihydroxyacetophenone, 1.5 mg mL^{-1} diammonium hydrogen citrate, 25% (*v/v*) ethanol, and 1% (*v/v*) trifluoroacetic acid on a 600 μm AnchorChip target (Bruker). An external mass calibration was done based on Protein Calibration Standard II (Bruker).

Methylene blue (MB) displacement assay. A calibration curve was first plotted against heparin concentration with the help of MB in PB. Fifty microliters of 10 μg mL^{-1} MB were mixed with 50 μL of heparin with different concentrations (0, 0.4, 0.8, 1.2, 1.6, and 2.0 μg mL^{-1}) in PB solutions. The absorption ratios (*A* (664 nm)/*A* (568 nm)) against heparin concentrations were plotted as the calibration curve (Figure S2a).

To determine the heparin-binding performance, different concentrations of PCSs or PS in PB were added to heparin solutions, yielding 100 μL PB solutions containing 2 μg mL^{-1} heparin and PCSs or PS in desired mass ratios (binder/heparin: 0×, 0.5×, 1×, 1.5×, 2×, 2.5×, 3×, 3.5×, 4×, 4.5× and 5× for PS; 0×, 3×, 6×, 9×, 12×, 15×, 18×, 21×, 24×, 27× and 30× for PCS0 and PCS4; 0×, 5×, 10×, 15×, 20×, 25×, 30×, 35×, 40×, 45× and 50× for PCS10). Then, 50 μL of the solution was added to a 50 μL MB solution (10 μg mL^{-1}) and the absorption spectra (450-750 nm) were recorded with a BioTek Cytation 3 microplate reader in a 96-well plate at room temperature. The absorbance intensity ratio *A* (664 nm) / *A* (568 nm) was used to determine the remaining heparin concentration in the solution and the heparin-binding ability was determined using the following equation:

$$\%_{\mathrm{Neutralization}} = [\ (c_0 - c_i)/c_0] \times 100$$

where c_0 is the derived heparin concentration at the mass ratio of 0 for all binders and c_i is the derived heparin concentration at the desired mass ratio. Measurements were performed using triplicate samples.

Supplementary Table and Figures

Table S1. Full amino acid sequences of the three PCSs.

**PCS0**
MetRFPSIFTAVLFAASSALAAPVNTTTEDETAQIPAEAVIGYSDLEGDFDVAVLPFSN<br>STNNGLLFINTTIASIAAKEEGVSLEKREAEAGPPGEPGNPGSPGNQGQPGNKGSPG<br>NPGQPGNEGQPGQPGQNGQPGEPGSNGPQGSQGNPGKNGQPGSPGSQGSPGNQGS<br>PGQPGNPGQPGEQGKPGNQGPAGEPGNPGSPGNQGQPGNKGSPGNPGQPGNEGQP<br>GQPGQNGQPGEPGSNGPQGSQGNPGKNGQPGSPGSQGSPGNQGSPGQPGNPGQPG<br>EQGKPGNQGPAGEPGNPGSPGNQGQPGNKGSPGNPGQPGNEGQPGQPGQNGQPG<br>EPGSNGPQGSQGNPGKNGQPGSPGSQGSPGNQGSPGQPGNPGQPGEQGKPGNQGP<br>AGEPGNPGSPGNQGQPGNKGSPGNPGQPGNEGQPGQPGQNGQPGEPGSNGPQGS<br>QGNPGKNGQPGSPGSQGSPGNQGSPGQPGNPGQPGEQGKPGNQGPAGGKKKKKK<br>KKKKKKG
**PCS4**
MetRFPSIFTAVLFAASSALAAPVNTTTEDETAQIPAEAVIGYSDLEGDFDVAVLPFSN<br>STNNGLLFINTTIASIAAKEEGVSLEKREAEAGCGLGAGAPGEPGNPGSPGNQGQP<br>GNKGSPGNPGQPGNEGQPGQPGQNGQPGEPGSNGPQGSQGNPGKNGQPGSPGSQ<br>GSPGNQGSPGQPGNPGQPGEQGKPGNQGPAGEPGNPGSPGNQGQPGNKGSPGNPG<br>QPGNEGQPGQPGQNGQPGEPGSNGPQGSQGNPGKNGQPGSPGSQGSPGNQGSPGQ<br>PGNPGQPGEQGKPGNQGPAGEPGNPGSPGNQGQPGNKGSPGNPGQPGNEGQPGQP<br>GQNGQPGEPGSNGPQGSQGNPGKNGQPGSPGSQGSPGNQGSPGQPGNPGQPGEQG<br>KPGNQGPAGEPGNPGSPGNQGQPGNKGSPGNPGQPGNEGQPGQPGQNGQPGEPGS<br>NGPQGSQGNPGKNGQPGSPGSQGSPGNQGSPGQPGNPGQPGEQGKPGNQGPAGA<br>GAGAGAGQGAGAGAGQGAGAGAGQGAGAGAGQGAKKKKKKKKKKKKGA
**PCS10**
MetRFPSIFTAVLFAASSALAAPVNTTTEDETAQIPAEAVIGYSDLEGDFDVAVLPFSN<br>STNNGLLFINTTIASIAAKEEGVSLEKREAEAGCGLGAGAPGEPGNPGSPGNQGQP<br>GNKGSPGNPGQPGNEGQPGQPGQNGQPGEPGSNGPQGSQGNPGKNGQPGSPGSQ<br>GSPGNQGSPGQPGNPGQPGEQGKPGNQGPAGEPGNPGSPGNQGQPGNKGSPGNPG<br>QPGNEGQPGQPGQNGQPGEPGSNGPQGSQGNPGKNGQPGSPGSQGSPGNQGSPGQ<br>PGNPGQPGEQGKPGNQGPAGEPGNPGSPGNQGQPGNKGSPGNPGQPGNEGQPGQP<br>GQNGQPGEPGSNGPQGSQGNPGKNGQPGSPGSQGSPGNQGSPGQPGNPGQPGEQG<br>KPGNQGPAGEPGNPGSPGNQGQPGNKGSPGNPGQPGNEGQPGQPGQNGQPGEPGS<br>NGPQGSQGNPGKNGQPGSPGSQGSPGNQGSPGQPGNPGQPGEQGKPGNQGPAGA<br>GAGAGAGQGAGAGAGQGAGAGAGQGAGAGAGQGAGAGAGQGAGAGAGQGA<br>GAGAGQGAGAGAGQGAGAGAGQGAGAGAGQGAKKKKKKKKKKKKGA
Color indication: blue: α-factor prepro secretory signal; green: the hydrophilic random-coil block “C”; pink: the silk-like sequence “S”; red: the heparin-binding segment “B”.

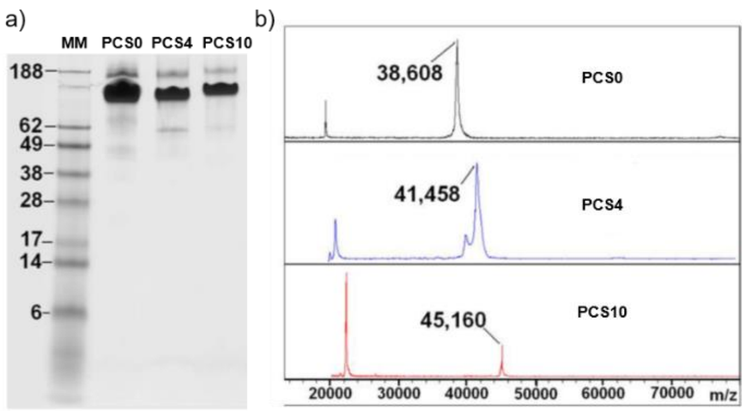

Figure S1. Characterization of PCSs. (a) SDS-PAGE image of PCSs, lane 1: PCS0, lane 2: PCS4, lane 3: PCS10, MM: molecular markers; (b) MALDI-TOF mass spectrum of PCSs displaying their measured molecular mass.

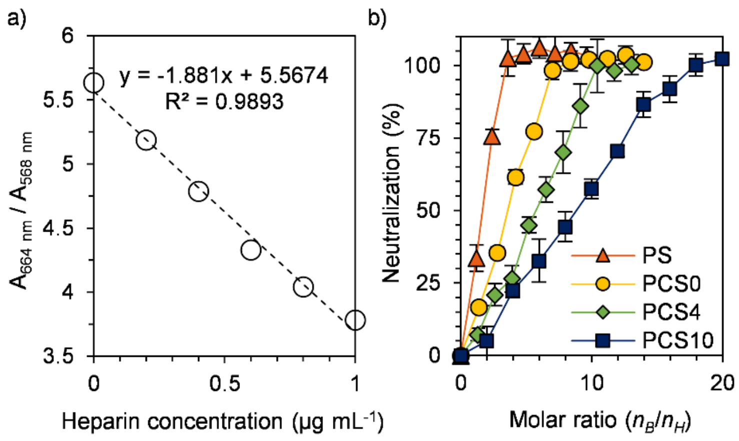

Figure S2. Heparin-neutralizing ability evaluated by MB-displacement assay. (a) Calibration curve of MB-displacement assay; (b) Evaluation of the heparin-neutralization ability in PB measured by MB-displacement assay.

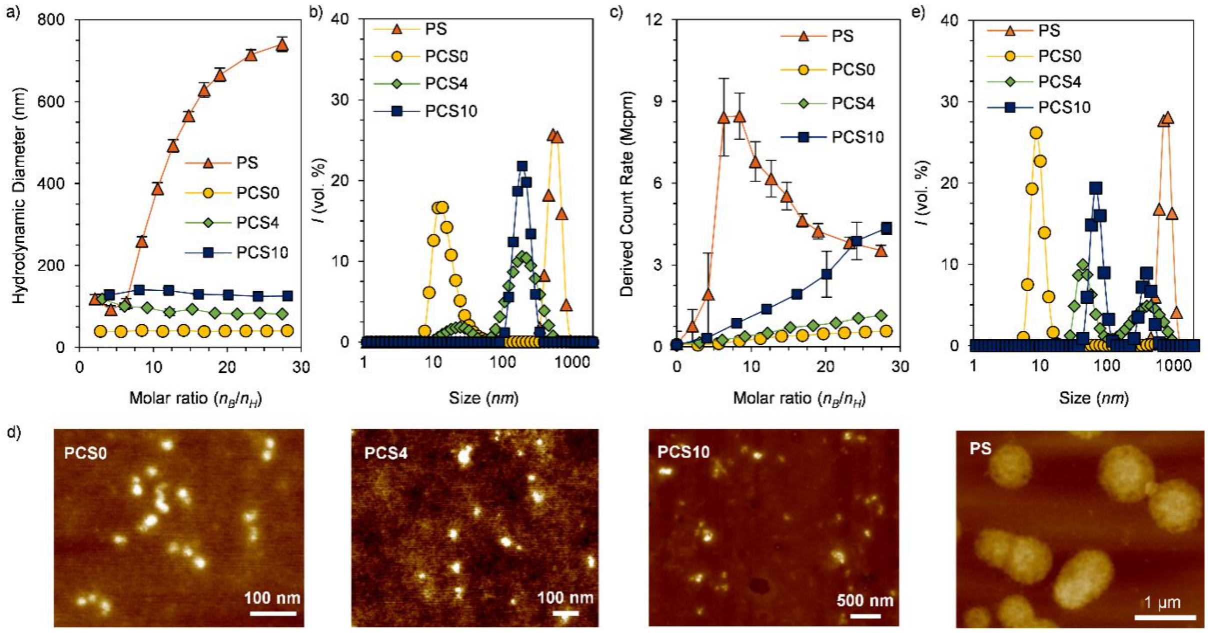

Figure S3. Evaluation of the heparin-neutralization ability of binders in PB and PBS. (a) The hydrodynamic diameters derived from DLS measurements through the titration of heparin solution (0.01 mg mL^{-1}) with binders in PB; (b) Representative volume proportions of binder-heparin complexes after the titration in PB; (c) The derived count rate obtained from DLS measurements through the titration of heparin solution (0.01 mg mL^{-1}) with binders in PB; (d) AFM images of the complexes formed by heparin and (from left to right) PCS0, PCS4, PCS10 and PS in PB; (e) Representative volume proportions of binder-heparin complexes after the titration in PBS.

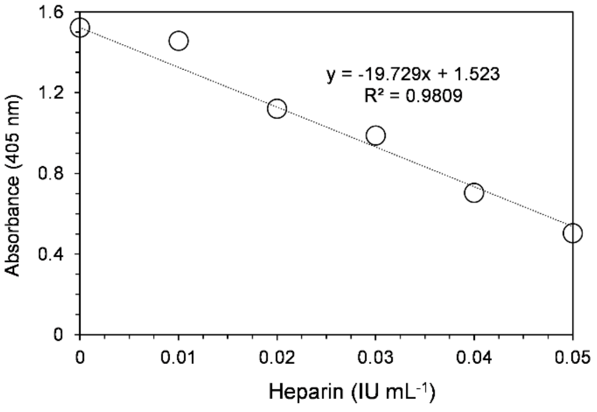

Figure S4. Calibration curve for the anti-Xa assay.

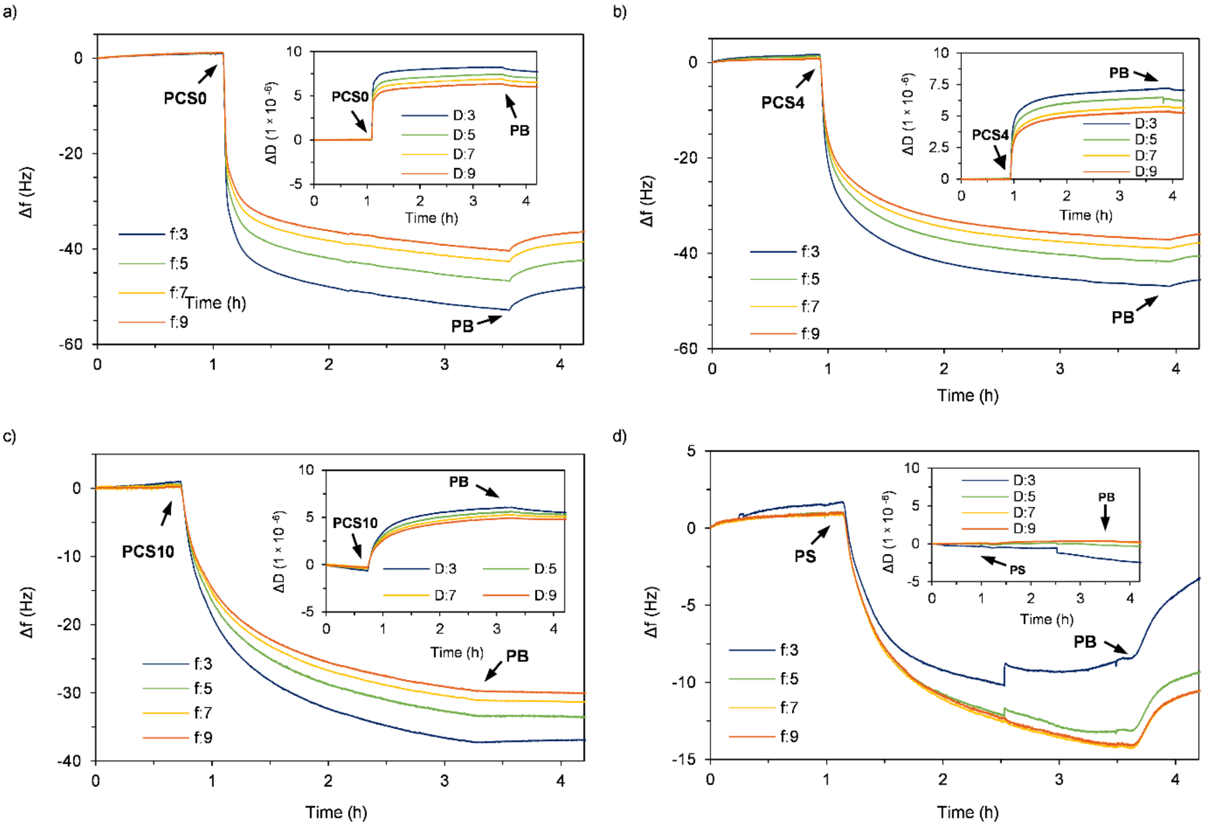

Figure S5. Frequency shifts (*Δf*) and energy dissipation (*ΔD*, insets) graphs from QCM-D measurements on the evaluation of PCS0 (a), PCS4 (b), PCS10 (c), and PS (d) binding heparin in PB.

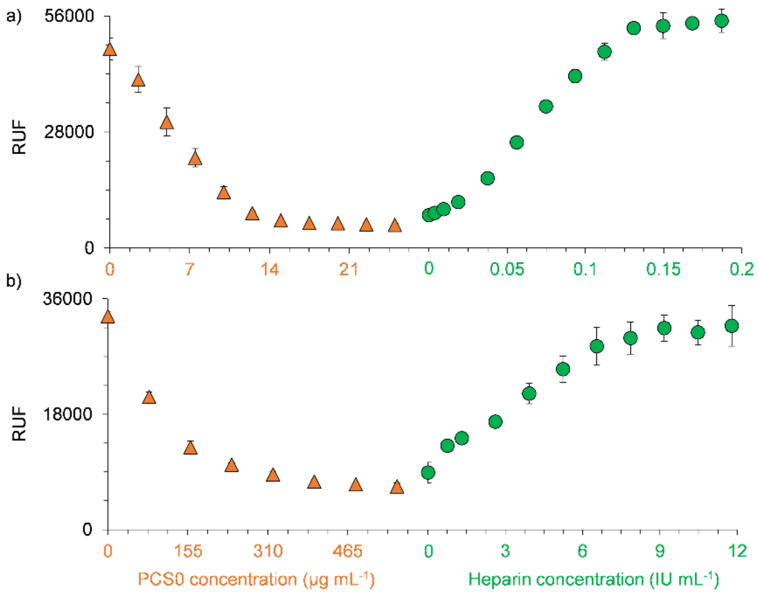

Figure S6. Fluorescence switch-on detection of heparin using PCS0 in PB (a) and PBS (b). Orange dots show the titration of 0.1 μM DNA with PCS0. Green dots show the fluorescence recovery upon adding heparin to the PCS0−DNA solution. Titrations were performed using triplicate samples, and the averaged results with standard deviation are presented.

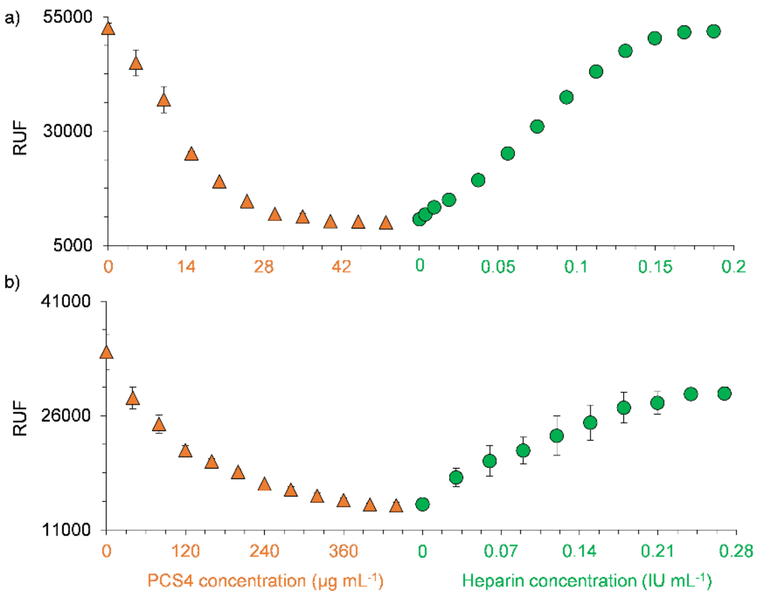

Figure S7. Fluorescence switch-on detection of heparin using PCS4 in PB (a) and PBS (b). Orange dots show the titration of 0.1 μM DNA with PCS4. Green dots show the fluorescence recovery upon adding heparin to the PCS4−DNA solution. Titrations were performed using triplicate samples, and the averaged results with standard deviation are presented.

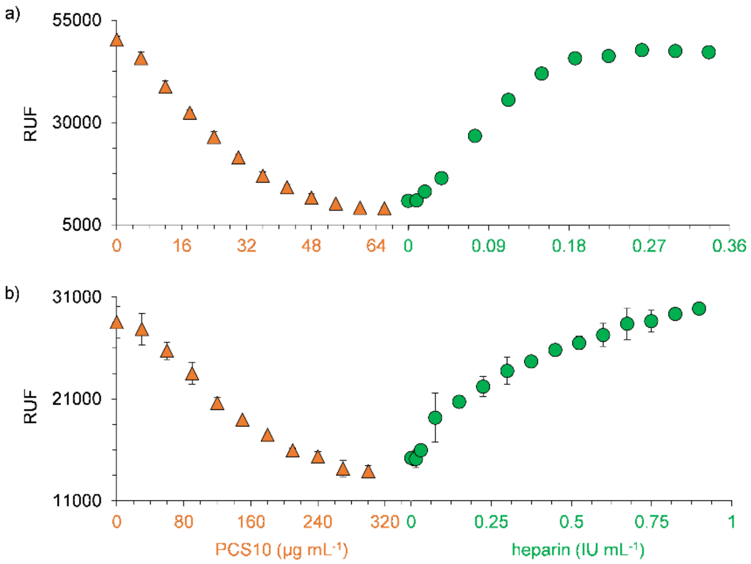

Figure S8. Fluorescence switch-on detection of heparin using PCS10 in PB (a) and PBS (b). Orange dots show the titration of 0.1 μM DNA with PCS10. Green dots show the fluorescence recovery upon adding heparin to the PCS10−DNA solution. Titrations were performed using triplicate samples, and the averaged results with standard deviation are presented.

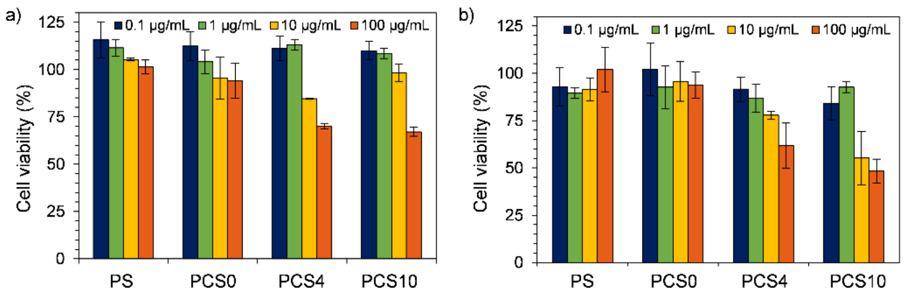

Figure S9. MTT assay of the incubation of human dermal fibroblasts (a) and Hep G2 (b) with binders for 24 h and 14 h, respectively. Cell cultures were performed using triplicate samples and the averaged results with standard deviation are presented.
